# Accuracy validation and user performance analysis of two new self-monitoring blood glucose systems

**DOI:** 10.1530/EDM-24-0134

**Published:** 2025-02-19

**Authors:** Tun-Yu Huang, Yu-Hsi Chen, Yu-Ching Li, Yen-Hsin Chiu, Hui-Jun Kang, Chih-Yu Chen

**Affiliations:** ^1^ Prospective Wound Medicine Research Center, Show Chwan Memorial Hospital, Changhua, Taiwan; ^2^ Department of Orthopedics, Show Chwan Memorial Hospital, Changhua, Taiwan; ^3^ Department of Family Medicine, Show Chwan Memorial Hospital, Changhua, Taiwan; ^4^ Microlife Medical Technology Co., Ltd, Taipei City, Taiwan; ^5^ Department of Orthopedics, Shuang-Ho Hospital, Taipei Medical University, New Taipei City, Taiwan; ^6^ International Ph.D. Program in Biomedical Engineering, College of Biomedical Engineering, Taipei Medical University, Taipei, Taiwan

**Keywords:** diabetes, self-glucose detection (monitoring) systems, user performance

## Abstract

**Summary:**

Patients with diabetes require regular blood glucose monitoring. We evaluated the precision and user performance of two blood glucose monitoring systems, the accuracy results of GlucoTeq BGM200 and DiaRite BGM300, adhering to the EN_ISO 15197:2015 standards, which require blood glucose ≥100 mg/dL, >95% of the data within ±15% difference, and when blood glucose <100 mg/dL, >95% of the data within ±15 mg/dL. This study assessed their conformity with the YSI analyzer and other leading systems. Participants (*n* = 101), 18 years of age were included, covering a diverse demographic. Accuracy was determined using the YSI analyzer as the standard, employing linear regression, a consensus error grid and Bland–Altman analyses. The performance of both system users showed 100% conformity within zone A of the consensus error grid, high linear regression coefficients and minimal bias in the Bland–Altman analysis. Subjective satisfaction analyses showed average scores of 4.59 and 4.62 for BGM200 and BGM300, respectively. Comparison with other systems revealed high regression coefficients (CONTOUR®PLUS; *R*^2^ = 0.9960, BGM200; *R*^2^ = 0.9927, BGM300; *R*^2^ = 0.9915, Accu-Chek® Guide; *R*^2^ = 0.9910). Both systems demonstrated reasonable accuracy and user performance comparable to competitors’ products and met international standards. Their reliability in real-world scenarios and high user satisfaction make them valuable tools for diabetes management.

**Learning points:**

- Reasonable accuracy: GlucoTeq BGM200 and DiaRite BGM300 complied with EN ISO 15197:2015 standards, achieving >95% accuracy validated against the reference method the YSI analyzer.
- High user satisfaction: both systems scored highly (4.59–4.62/5), reflecting ease of use, reliable operation and clear result interpretation.
- Competitive performance: comparable to leading devices such as CONTOUR®PLUS, with strong correlation coefficients (>0.99).
- Regulatory compliance: the trial met stringent international standards with a diverse participant group and robust methodology.
- Broad applicability: reliable for clinical and home use, supporting effective diabetes management.

## Background

Diabetes, a chronic condition affecting over 537 million people globally, requires consistent management to prevent complications such as cardiovascular disease and kidney damage (1, 2, 3). Regular blood glucose monitoring is crucial for assessing treatment effectiveness and guiding therapeutic interventions. This study evaluated two advanced self-monitoring systems, GlucoTeq BGM200 and DiaRite BGM300, designed to meet EN ISO 15197:2015 standards (4, 5, 6, 7). By providing accurate and user-friendly tools, these systems aim to enhance patient engagement in diabetes care and improve outcomes, addressing a critical need in global health management.

## Study design

This study evaluated the accuracy validation and user performance of two self-monitoring blood glucose systems, GlucoTeq BGM200 and DiaRite BGM300, using rigorous methods to meet EN ISO 15197:2015 standards. The investigation included 101 participants representing a diverse demographic profile (8).

### Accuracy validation test

The systems were assessed by professionals for accuracy validation against the YSI 2300 biochemical analyzer, the reference method for blood glucose measurements. In addition, participants used two glucose systems by themselves and completed questionnaires after self-testing blood from both fingertip and non-fingertip sites. Results showed that both systems performed well, with compliance rates over 95% for all glucose ranges and minimal bias detected through Bland–Altman analysis. In addition, linear regression analyses revealed high correlation coefficients (*R*^2^ > 0.99), while consensus error grid evaluations confirmed that 100% of the measurements fell within the clinically acceptable zone A.

### User performance test

Beyond accuracy, the study focused on user performance and satisfaction. Participants independently used the devices and rated their usability and ease of operation. Satisfaction scores were high, averaging 4.59 for GlucoTeq and 4.62 for DiaRite, reflecting user-friendly designs and clear instructions. These findings confirm that both devices are highly accurate, reliable and practical for home and clinical use, making them valuable tools for improving diabetes self-management and patient care outcomes.

## Methods

### Accuracy validation test

**Figure 1:**
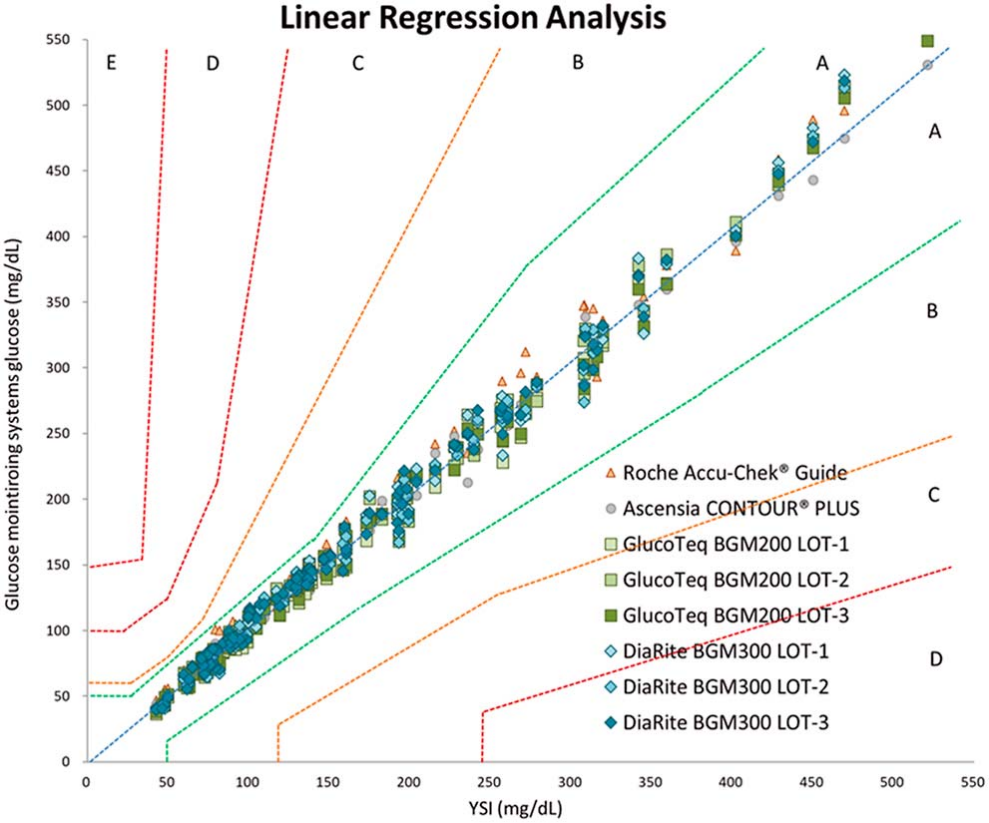
Combined analyzes result of the consensus error grid and linear regression between the YSI analyzer and the GlucoTeq BGM200 and DiaRite BGM300 blood glucose monitoring systems, operated by medical technologists. The test results of both systems demonstrated 100% conformity within zone A. Furthermore, the regression coefficient results demonstrated that both systems demonstrated consistently high correlation with the YSI analyzer (GlucoTeq BGM200 *R*^2^ = 0.9927 and DiaRite BGM300 *R*^2^ = 0.9916, respectively).

**Figure 2:**
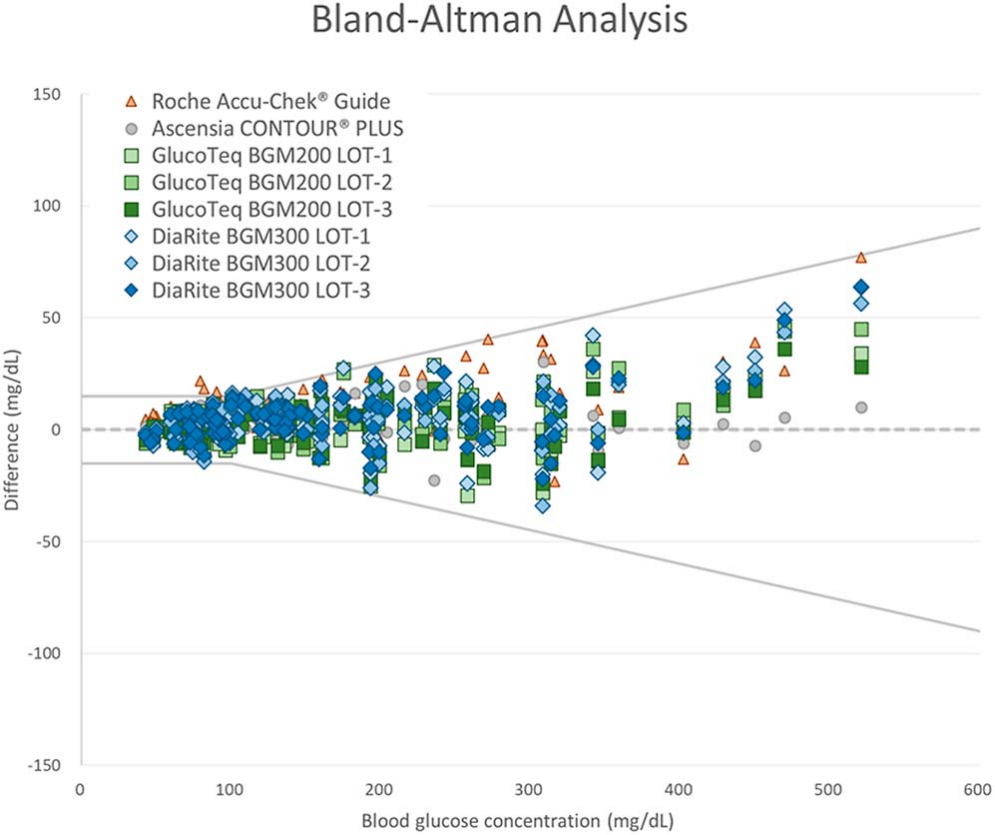
Bland–Altman analysis comparing GlucoTeq BGM200, DiaRite BGM300, Ascensia CONTOUR^®^ PLUS and Roche Accu-Chek^®^ Guide against the YSI 2300 analyzer.

The accuracy validation (performed by professionals) of the GlucoTeq BGM200 and DiaRite BGM300 blood glucose monitoring systems demonstrated their accuracy and reliability, adhering to EN ISO 15197:2015 standards. Using the YSI 2300 analyzer as the reference method, both devices achieved over 95% compliance within acceptable accuracy ranges, with GlucoTeq showing 99.7% and DiaRite 98.8% compliance. Statistical analyses, including linear regression (*R*^2^ > 0.99 for both devices) (Fig. 1), consensus error grid (100% measurements in zone A) and Bland–Altman analysis (Fig. 2), confirmed strong agreement with the reference standard (8). These results underscore the clinical precision of the devices across a broad range of glucose levels, including high-risk categories below 100 mg/dL. In addition, another two commercially available blood sugar monitoring systems (Ascensia CONTOUR^®^PLUS (Switzerland) and Roche Accu-Chek^®^ Guide (Switzerland)) were also tested following same protocol.

### User performance test

**Figure 3:**
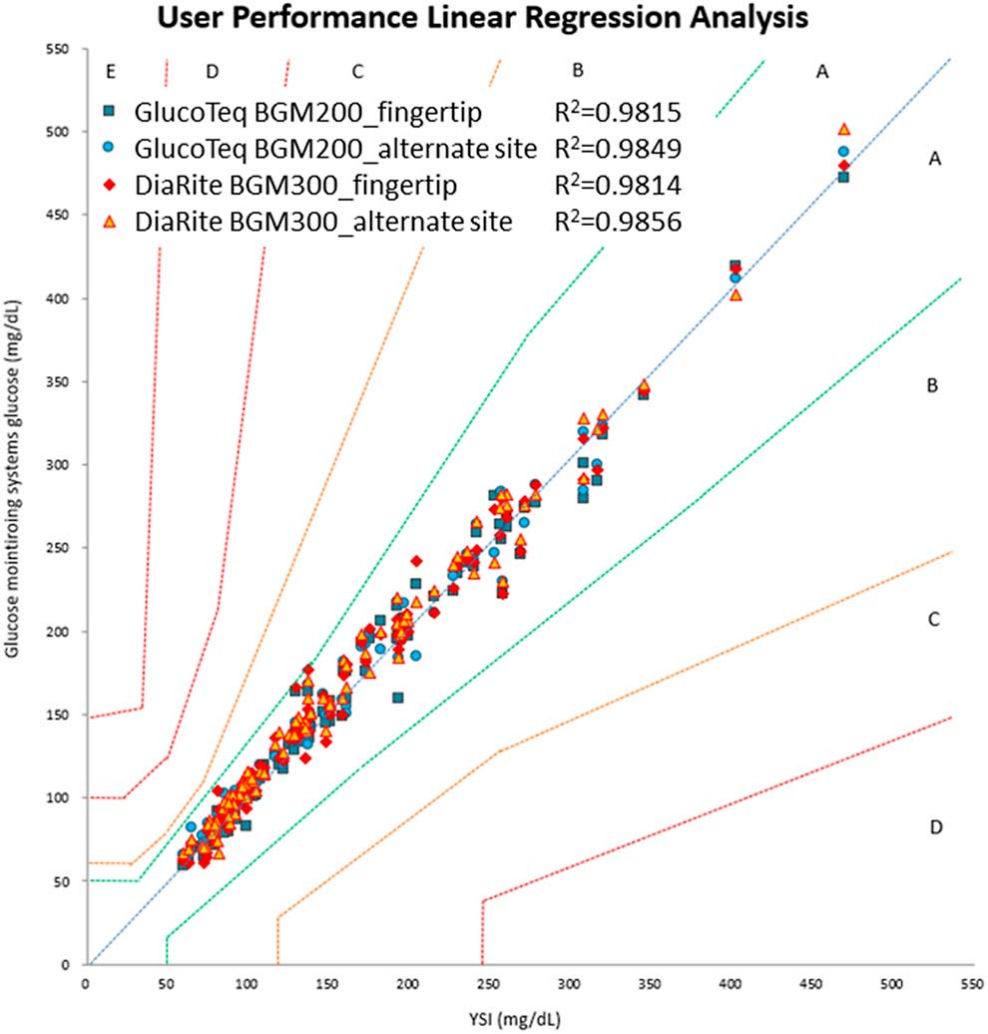
Results of the linear regression of the performance of the user-operated GlucoTeq BGM200 and DiaRite BGM300 blood glucose monitoring systems at the fingertip and at the alternate site (non-fingertip), respectively. All test results fell within zone A of the consensus error grid. The regression coefficient analysis revealed a strong and consistent correlation with the YSI analyzer, as indicated by a coefficient of determination (*R*^2^) exceeding 0.95 in both systems (GlucoTeq BGM200 had *R*^2^ = 0.9815 at fingertip, *R*^2^ = 0.9849 at alternate site and DiaRite BGM300 had *R*^2^ = 0.9814 at fingertip, *R*^2^ = 0.9856 at alternate site).

**Figure 4:**
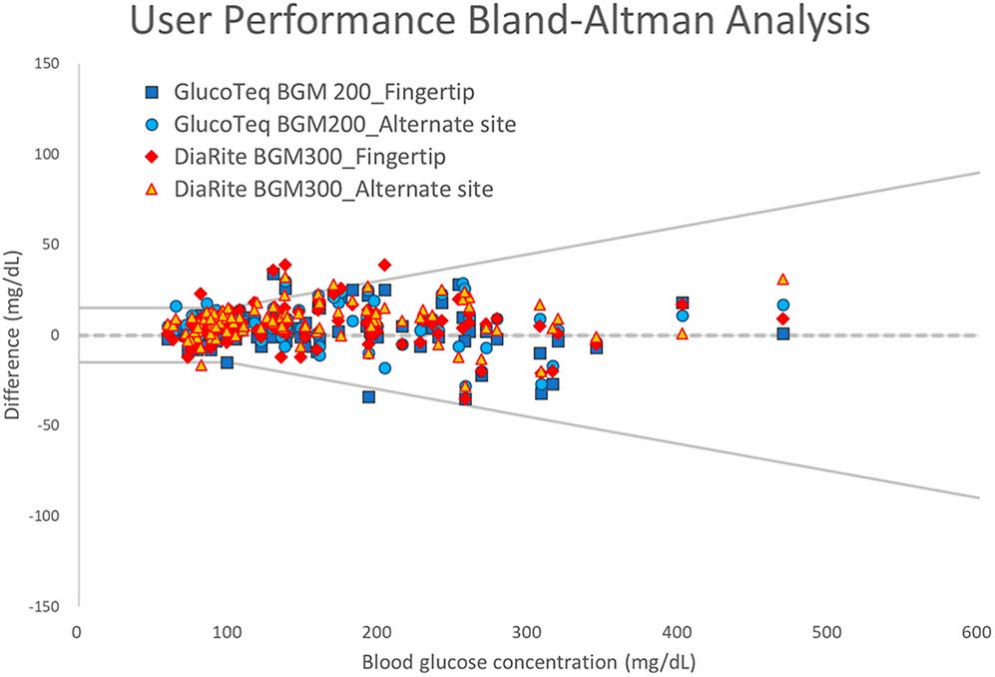
Bland–Altman analysis of GlucoTeq BGM200 and DiaRite BGM300 blood glucose monitoring systems operated by participants at the fingertip and alternate site (non-fingertip).

User performance (lay user) testing highlighted the devices’ usability, with participants achieving similar accuracy without professional intervention (Figs 3 and 4 and Table 1). Satisfaction evaluations reflected positive experiences, with GlucoTeq scoring 4.59/5 and DiaRite 4.62/5 for setup, operation and result interpretation (Table 2).

**Table 1:** User performance evaluation of blood glucose concentration intervals based on YSI analyzer measured value.

	GlucoTeq BGM200*			DiaRite BGM300^†^		
	Value within ±5 mg/dL	Value within ±10 mg/dL	Value within ±15 mg/dL	Value within ±5 mg/dL	Value within ±10 mg/dL	Value within ±15 mg/dL
Fingertip measurement						
Blood glucose lower than 100 mg/dL	21/37 (56.8%)	35/37 (94.6%)	36/37 (97.3%)	18/37 (48.6%)	33/37 (89.2%)	37/37 (100%)
Blood glucose higher or equal to 100 mg/dL	41/64 (64.1%)	53/64 (82.8%)	64/64 (100.0%)	31/64 (48.4%)	52/64(81.3%)	62/64 (96.9%)
Alternate site (non-fingertip) measurement						
Blood glucose lower than 100 mg/dL	13/37 (35.1%)	33/37 (89.2%)	37/37 (100%)	12/37 (32.4%)	25/37 (67.6%)	36/37 (97.3%)
Blood glucose higher or equal to 100 mg/dL	37/64 (57.8%)	51/64 (79.7%)	64/64 (100.0%)	26/64 (40.6%)	53/64 (82.8%)	61/64 (95.3%)

* GlucoTeq BGM200 measure value with all blood glucose value, based on EN ISO15197:2015 standard specification value within ±15 mg/dL (±0.83 mmol/L) or ±15% 100/101(99.0%).

^†^ DiaRite BGM200 measure value with all blood glucose value, based on EN ISO15197:2015 standard specification value within ±15 mg/dL (±0.83 mmol/L) or ±15% 99/101(98.0%) for fingerprick measurement and 97/101(96.0%) for non fingerprick measurement.

**Table 2:** Satisfaction analyses of GlucoTeq BGM200 and DiaRite BGM300 blood glucose monitoring systems.

Events	Score	
	GlucoTeq BGM200	DiaRite BGM300
Setting		
Install batteries	4.64	4.66
Setting date and time	4.32	4.43
Measurement of blood glucose		
Insert the test strip	4.70	4.72
Fingerstick	4.64	4.71
Apply blood sample	4.71	4.70
Remove the strip	4.70	4.74
Results displayed		
Read the results	4.73	4.73
Understand the results	4.68	4.73
Description of operating manual		
The manual is easy to follow	4.42	4.52
The operating manual clearly explain what to do when error messages displayed	4.45	4.49
Understand how the AC/PC function setting		4.47
Average score	4.59	4.62

The satisfaction rate is assessed using the Likert five-point scoring method, where ‘Very poor’ corresponds to 1 point, ‘Poor’ to 2 points, ‘Fair’ to 3 points, ‘Good’ to 4 points and ‘Very good’ to 5 points. A higher score indicates ease of reading or operation.

## Results

### Accuracy validation results

Figures 1 and 2 illustrated the results of ‘accuracy validation’ for two systems. The linear regression and Bland–Altman analyses in these figures highlighted the agreement and correlation between the systems and the reference method, demonstrating that both devices achieved high accuracy across different glucose concentration ranges. Both systems maintained high accuracy, with compliance rates complied with EN ISO 15197:2015 standards.

### User performance results

On the other hand, Figs 3 and 4 and Table 1 demonstrated the results of the ‘user performance (lay user) test’, where participants independently operated the two systems. This test involved self-collecting blood samples from the fingertip and alternate sites (non-fingertip areas such as the palm, forearm or thigh). The performance metrics presented in Figs 3 and 4 reflected the usability and accuracy of the systems when operated by non-professionals in real-world conditions. Figures 3 and 4 showed that lay users could achieve results comparable to those obtained by trained professionals (Figs 1 and 2), further confirming the user-friendliness and reliability of the devices. Post-evaluation satisfaction analysis (Table 2) demonstrated consistently high satisfaction scores (averaging 4.59 and 4.62 for GlucoTeq and DiaRite, respectively), reflecting reliable operation in real-world settings. No adverse effects or significant usability issues were reported, and the devices demonstrated robust performance across the follow-up period, confirming their suitability for both clinical and home settings in managing diabetes.

## Discussion

Accurate monitoring of blood glucose is critical, especially for people who require frequent insulin adjustments to maintain stable blood glucose levels and prevent hypoglycemia (9, 10). Our meticulous evaluation of the GlucoTeq and DiaRite systems, adhering strictly to the EN ISO 15197: 2015 standard specification, revealed that more than 95% of the collected data fell within the predefined acceptable range, validated by the Bland–Altman comparison method, demonstrating a strong alignment with the reference method, the YSI analyzer. Specifically, data corresponding to blood glucose concentrations below 100 mg/dL demonstrated compliance within a range of ±15 mg/dL, highlighting precision and consistency in accurately measuring lower glucose concentrations. In contrast, for blood glucose concentrations equal to or greater than 100 mg/dL, these systems maintained a bias within ±15%, reaffirming their reliability and clinical precision of these systems across a broad spectrum of glucose levels. These findings emphasize the indispensable role of these two systems in effective glucose monitoring, positioning them as reliable tools in various healthcare settings. In addition, both the GlucoTeq and DiaRite systems showed evenly matched results when compared with other well-known blood glucose monitoring systems.

Furthermore, our user performance evaluation effectively validated the practical accuracy and reliability of the GlucoTeq and DiaRite systems in real-world applications for individuals lacking specialized training. The remarkable alignment of glucose values obtained by subjects with the consensus standards outlined in EN ISO 15197:2015, for both fingertip and alternate-site testing strongly supports the clinical precision of these systems when operated by lay users. The results of the user performance analysis validated the effectiveness and precision of the two systems tested in different user scenarios and reinforced the confidence that GlucoTeq and DiaRite are highly reliable and suitable for use in diverse settings. In addition, the subjective satisfaction questionnaire demonstrated 4.59 and 4.62 out of 5 points for both systems.

In summary, both devices are highly accurate, reliable and practical for home and clinical use, making them valuable tools for improving diabetes self-management and patient care outcomes.

## Declaration of interest

YHChiu and HUK were employees of Microlife medical technology assigned to assist with this study, and both declared that they have no financial conflict of interest in this study other than the salary for which they were employed. All other authors declare that they have neither financial nor non-financial conflict of interest with the publication of this clinical trial.

## Funding

This study was fully funded by Microlife Medical Technology Co., Ltd, Taipei City, Taiwan. This grant includes experimental equipment and biochemical testing fees, participant recruitment fees, IRB committee and institutional research management and administrative processing fees and article processing charges (APCs). In addition, YHChiu and HUK were employees of Microlife assigned to assist with this study.

## Patient consent

The study was conducted in accordance with the Declaration of Helsinki and approved by the Institutional Review Board of Show Chwan Memorial Hospital (SCMH_IRB no. 1120601).

Written consent has been obtained from each patient after full explanation of the purpose and nature of all procedures used.

## Author contribution statement

TYH, YHChen, YCL and THT designed the study. YHChen, HUK, THT and CYC designed the methodology. TYH, YHChen, YCL and CYC validated the method. TYH, YHChiu and YCL performed the formal analysis. YCL obtained the resources. TYH, YHChiu and YCL performed data curation. CYC prepared the original draft. CYC reviewed and edited the manuscript. TYH, YHChen, YCL and CYC contributed to visualization. YHChen, HUK and CYC supervised the study. YHChen, HUK and CYC contributed to project administration. YHChen, HUK and CYC assisted in funding acquisition. All authors have read and agreed to the final version of the manuscript.
